# A novel artificial intelligence model for fetal facial profile marker measurement during the first trimester

**DOI:** 10.1186/s12884-023-06046-x

**Published:** 2023-10-10

**Authors:** Chunya Ji, Kai Liu, Xin Yang, Yan Cao, Xiaoju Cao, Qi Pan, Zhong Yang, Lingling Sun, Linliang Yin, Xuedong Deng, Dong Ni

**Affiliations:** 1 Center for Medical Ultrasound, Suzhou Municipal Hospital, Gusu School, The Affiliated Suzhou Hospital of Nanjing Medical University, Nanjing Medical University, Suzhou, Jiangsu China; 2 National-Regional Key Technology Engineering Laboratory for Medical Ultrasound, School of Biomedical Engineering, Health Science Center, Shenzhen University, Xueyuan Blvd, Nanshan, Shenzhen, Guangdong China; 3 Shenzhen RayShape Medical Technology Co., Ltd, Shenzhen, Guangdong China; 4 Center for Reproduction and Genetics, Suzhou Municipal Hospital, Gusu School, The Affiliated Suzhou Hospital of Nanjing Medical University, Nanjing Medical University, No. 26 Daoqian Street, Suzhou, 215002 Jiangsu China

**Keywords:** Artificial intelligence, First trimester, Abnormal screen, Fetus, Facial profile, Markers, Deep learning, Prenatal ultrasonography, Automatic measurement

## Abstract

**Background:**

To study the validity of an artificial intelligence (AI) model for measuring fetal facial profile markers, and to evaluate the clinical value of the AI model for identifying fetal abnormalities during the first trimester.

**Methods:**

This retrospective study used two-dimensional mid-sagittal fetal profile images taken during singleton pregnancies at 11–13^+ 6^ weeks of gestation. We measured the facial profile markers, including inferior facial angle (IFA), maxilla-nasion-mandible (MNM) angle, facial-maxillary angle (FMA), frontal space (FS) distance, and profile line (PL) distance using AI and manual measurements. Semantic segmentation and landmark localization were used to develop an AI model to measure the selected markers and evaluate the diagnostic value for fetal abnormalities. The consistency between AI and manual measurements was compared using intraclass correlation coefficients (ICC). The diagnostic value of facial markers measured using the AI model during fetal abnormality screening was evaluated using receiver operating characteristic (ROC) curves.

**Results:**

A total of 2372 normal fetuses and 37 with abnormalities were observed, including 18 with trisomy 21, 7 with trisomy 18, and 12 with CLP. Among them, 1872 normal fetuses were used for AI model training and validation, and the remaining 500 normal fetuses and all fetuses with abnormalities were used for clinical testing. The ICCs (95%CI) of the IFA, MNM angle, FMA, FS distance, and PL distance between the AI and manual measurement for the 500 normal fetuses were 0.812 (0.780–0.840), 0.760 (0.720–0.795), 0.766 (0.727-0.800), 0.807 (0.775–0.836), and 0.798 (0.764–0.828), respectively. IFA clinically significantly identified trisomy 21 and trisomy 18, with areas under the ROC curve (AUC) of 0.686 (95%CI, 0.585–0.788) and 0.729 (95%CI, 0.621–0.837), respectively. FMA effectively predicted trisomy 18, with an AUC of 0.904 (95%CI, 0.842–0.966). MNM angle and FS distance exhibited good predictive value in CLP, with AUCs of 0.738 (95%CI, 0.573–0.902) and 0.677 (95%CI, 0.494–0.859), respectively.

**Conclusions:**

The consistency of fetal facial profile marker measurements between the AI and manual measurement was good during the first trimester. The AI model is a convenient and effective tool for the early screen for fetal trisomy 21, trisomy 18, and CLP, which can be generalized to first-trimester scanning (FTS).

## Background

Fetal facial abnormalities, such as cleft lip and palate (CLP) and micrognathia, are associated with structural abnormalities in other systems and genetic syndromes [1, 2]. These facial abnormalities inflict considerable distress on affected children and their families and impose a severe burden on society as a whole. Therefore, the early screen for facial abnormalities is of particular importance. Ultrasonography is first line for fetal facial structure screening as images are available in real-time, no radiation exposure and results are replicable. Facial markers during the first trimester, such as inferior facial angle (IFA), maxilla-nasion-mandible (MNM) angle, facial-maxillary angle (FMA), frontal space (FS) distance, and profile line (PL) distance, can be measured using ultrasonography. Studies have found that abnormalities in these markers can indicate fetal facial deformities (such as CLP and micrognathia) or genetic abnormalities (such as trisomy 21 and trisomy 18) [2–6]. In clinical practice, it took approximately 5–6 min to measure all the five markers three times. The traditional manual measurement method is time-consuming, and requires sonographers with exceptional expertise in fetal medicine and practical experience. Consequently, conducting effective assessments in primary hospitals is difficult.

In recent years, research on the role of deep learning (DL) technology in the field of fetal ultrasound has increased. DL has proven to be an efficient tool for medical image-processing tasks by automatically extracting semantic features from images, [7, 8] applications in prenatal ultrasound include object detection, [9, 10] semantic segmentation, [11–13] and landmark localization [14]. Sun et al. [15] proposed the Least Absolute Shrinkage and Selection Operator (LASSO) method, which incorporates fetal nuchal translucency (NT) thickness, along with various facial profile markers, including pre-nasal thickness (PT) and MNM angle. It can serve as an efficient prognostic method for trisomy 21 during the first trimester.

This study aimed to develop an artificial intelligence-based measurement model for facial profile markers, including IFA, MNM angle, FMA, FS distance, and PL distance. We then aimed to assess its validity and diagnostic efficacy for fetal abnormalities, such as trisomy 21, trisomy 18, and CLP, in the first trimester.

## Methods

### Subjects

This retrospective study utilized archived fetal images of singleton pregnancies acquired through first trimester scanning (FTS) at the Affiliated Suzhou Hospital of Nanjing Medical University, Suzhou, China, between January 2020 and March 2022. We selected two-dimensional images from the mid-sagittal plane of the fetal face at 11–13^+ 6^ weeks of gestation. The inclusion criteria were as follows: (1) the mid-sagittal image of the fetal face showed only the head and upper chest, with the fetal head occupying more than 75% of the screen; (2) fetal facial structures, including the forehead, nasal bone, nasion, palate, mandible, chin, and upper/lower lip, were clearly displayed in the image; (3) fetal facial images were unobstructed by the umbilical cord or fetal limbs; and (4) singleton pregnancy with follow-up results. The exclusion criteria were as follows: (1) blurred images resulting in an unclear fetal facial structure, and (2) fetuses lost to follow-up. All acceptable images should meet inclusion criteria and exclusion criteria. This study was approved by the Ethics Committee of Suzhou Municipal Hospital (K-2022-011-K01). All pregnant women signed informed consent forms.

**Fig. 1:**
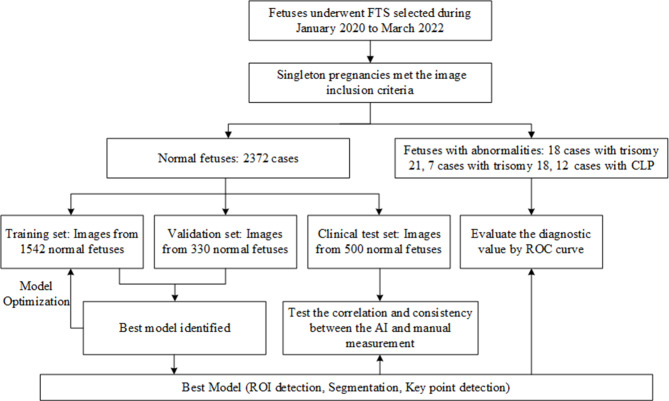
Flowchart summarizing the study design

A flowchart summarizing the development and validation of the AI model is shown in Fig. 1. We excluded 312 normal fetuses based on inclusion and exclusion criteria. A total of 2372 normal fetuses (median (interquartile range (IQR)) maternal age, 29 (27–31) years) and 37 fetuses with abnormalities (median (IQR) maternal age, 31 (28–35) years) were selected, including 18 with trisomy 21, 7 with trisomy 18, and 12 with CLP. The average width and height of all images are 1027.85 and 745.48, respectively. Within the normal fetal group, 500 fetuses were randomly selected as the clinical test set, and the additional fetuses was divided in an 8:2 ratio into a training set (1542 fetuses) and a validation set (330 fetuses). Within the abnormal fetal group, all fetuses with abnormalities were subjected to clinical testing to confirm the diagnostic efficacy of the fetal facial markers. In the clinical test set of normal fetuses, all markers were measured using the AI model (AI group) and by a senior sonographer (manual measurement group) who had obtained the NT screening qualification certificate granted by the Fetal Medicine Foundation (FMF). The manual measurement results were scrutinized by another experienced sonographer certified by the FMF.

### Equipment, software and quality control

In this study, Philips Affiniti70 four-dimensional (4D) color ultrasound diagnostic equipment with C9-2 two-dimensional probes (2–9 MHz frequency range) was employed. The transabdominal ultrasound images were imported in JPG format into the medical image intelligent software Pair [16] (version 2.6; Shenzhen, China), developed by Shenzhen RayShape Medical Technology Co., Ltd.

Each standard section of the FTS was annotated by an experienced senior sonographer certified in FMF and subsequently evaluated by another FMF-certified sonographer of the same caliber. The two-dimensional ultrasound (2D-US) scanning was executed in strict accordance with the guidelines provided by the International Society of Ultrasound in Obstetrics and Gynecology (ISUOG) [17] as well as FMF. The fetal crown-rump length (CRL) and NT thickness were measured, and the fetal nasal bone evaluated. Gross structure scanning, included the fetal head, face, spine, heart, thoracic/abdominal cavity, thoracic/abdominal wall, kidney, bladder, and limbs, was completed. Additionally, fetal appendages, such as the placenta, amniotic fluid, umbilical cord, and cervix, were observed.

### Markers and annotations

**Fig. 2:**
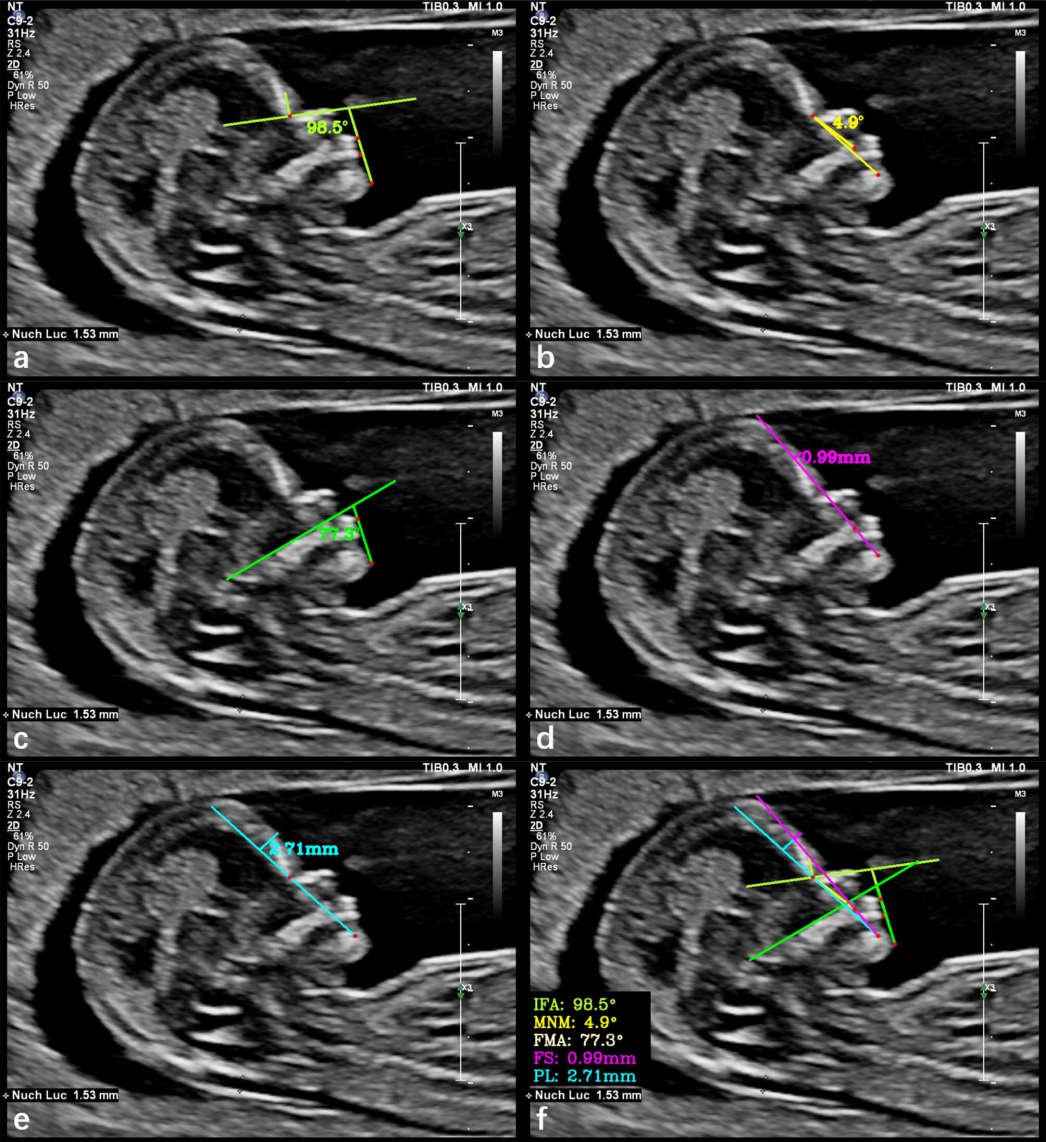
Ultrasound images showing the manual measurement of each facial profile markers: **(a)** inferior facial angle (IFA), 98.5°; **(b)** maxilla-nasion-mandible (MNM) angle, 4.9°; **(c)** facial-maxillary angle (FMA), 77.3°; **(d)** frontal space (FS) distance, 0.99 mm; **(e)** profile line (PL) distance, 2.71 mm; **(f)** summary graph of all markers

The following profile markers were measured in our study:

1. IFA [3, 18]: Angle between the line orthogonal to the vertical part of the forehead at the level of the synostosis of the nasal bones and a second line joining the tip of the mentum to the anterior point of the more protruding lip (Fig. 2a).
2. MNM angle [4, 18]: the angle between the maxillary and mandibular nasion lines (Fig. 2b). The nasion [5] was defined as the most anterior point at the intersection of the frontal and nasal bones.
3. FMA [2, 18]: Angle between the line overlying the maxilla and the line across the mentum tip and upper lip (Fig. 2c).
4. FS distance [6, 18]: Maximum perpendicular distance from the mandibular-maxillary line (MML) to the most prominent part of the fetal forehead (Fig. 2d). The MML is an extended line that intersects the most anterior portions of the mandible and maxilla. When the MML was located anterior to the forehead, the distance was multiplied by -1.
5. PL distance [5, 18]: The maximum perpendicular distance from the facial profile line (FPL) to the outer border of the forehead (Fig. 2e). The FPL is the line passing through the midpoint of the anterior border of the mandible and nasion. A summary diagram of all measured markers were shown in Fig. 2f.

To train the AI model, a specialized FMF-certified sonographer manually annotated all images in the training and validation sets. Altogether, we annotated four anatomical structures that required segmentation, including the fetal forehead, maxilla, mandible, and tangent at the nasion, as well as six crucial landmarks that required localization, including the upper/lower lip, middle point of the anterior border of the maxilla/mandible, mentum, and nasion. The above annotation content was reviewed by an additional senior FMF-certified sonographer to ensure the accuracy of the labeling results.

### AI model architecture

**Fig. 3:**
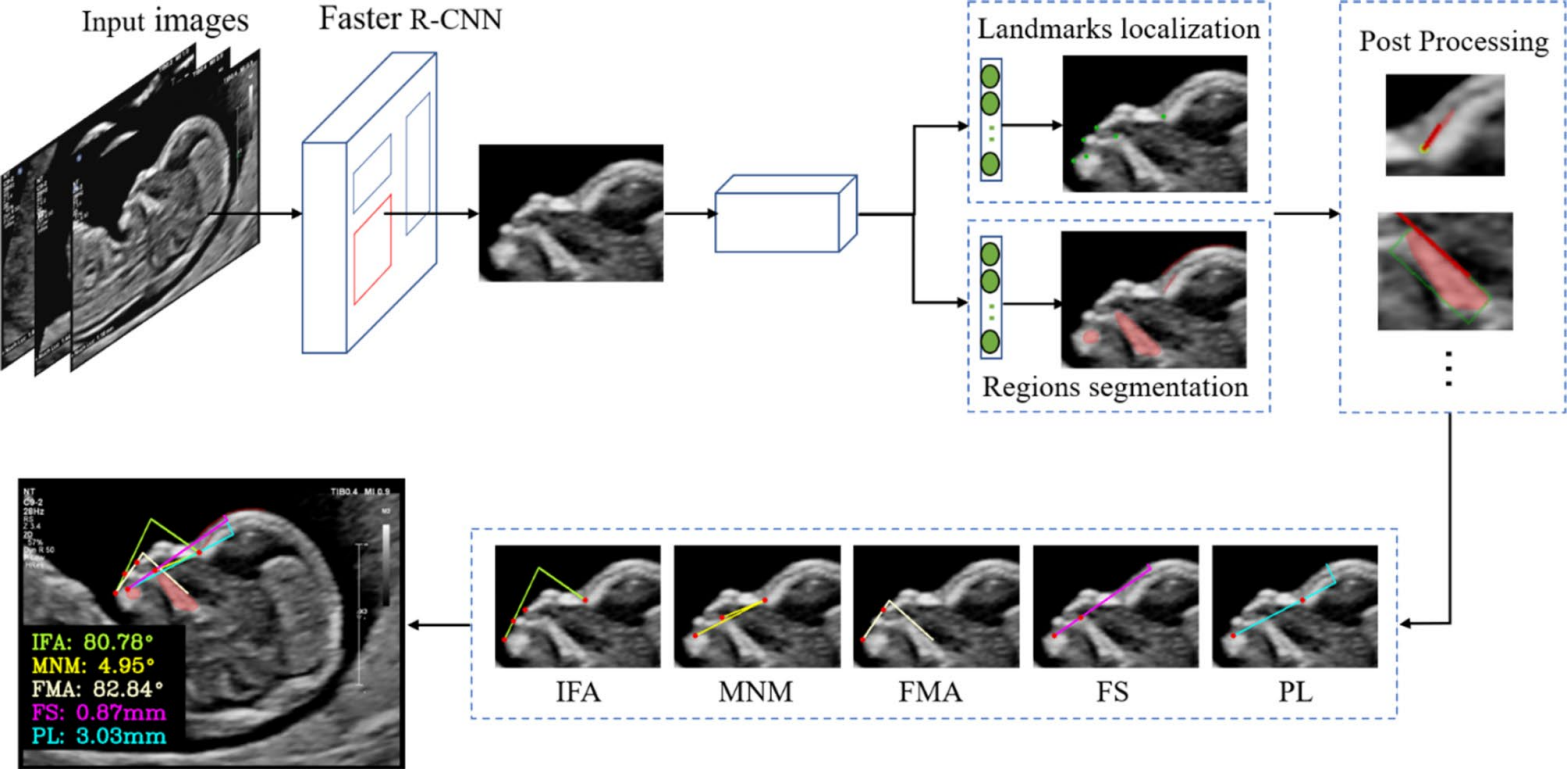
Flowchart illustrating the measurement process of facial profile markers by AI model during the first trimester

As illustrated in Fig. 3, the images of the mid-sagittal section were initially processed using Faster-RCNN [19] to acquire the region of interest (ROI), which contained anatomical information required for segmentation and localization. In order to expand the diversity of data sets and enhance the robustness of the model, the ROI is further expanded by a variety of data enhancement methods [20], such as grayscale processing, random scaling, random flipping, random rotation, etc.

Next, convolutional neural networks [21] (CNNs) were used to extract multiscale semantic features of key anatomical structure. These features were then outputted through the parallel multitask branch network to obtain the regional segmentation of the four crucial anatomical structures (forehead, maxilla, mandible, and tangent at the nasion), and the location prediction results of six anatomical landmarks (upper/lower lip, middle point of the anterior border of the maxilla/mandible, mentum, and nasion). The set similarity measure function (Dice) loss [22] and the mean square of structure point (MSE) loss function will be used to constrain the training of the segmentation task and the key point localization task network, respectively.

Based on the above results, the landmarks of the anatomical structures were optimized by post-processing, and relevant marker measurements were calculated. Finally, the model restored the result to the corresponding position in the original image, according to the location of the ROI.

### Statistical analysis

SPSS (version 22.0; Chicago, IL, USA) and GraphPad Prism (version 8.2; San Diego, CA, USA) software were used for the statistical analysis. We used the average Euclidean distance to evaluate the error between the predicted and actual landmarks as labeled by the sonographer. Continuous variables with a Gaussian distribution are expressed as mean (standard deviation, SD). Continuous variables without a Gaussian distribution are expressed as medians (interquartile range, IQR). Pearson correlation test was used to analyze the differences between the AI group and manual measurement group, and a P value less than 0.05 was deemed statistically significant. Consistency between the two groups was compared using the intraclass correlation coefficient (ICC, “Two-way random”, “absolute agreement”) and Bland–Altman analysis [23]. Finally, we calculated the AUC and 95% Confidence Interval (95%CI) to ascertain the diagnostic value of the AI model for identifying fetal abnormalities.

## Results

### Performance of AI measurement model for fetal facial markers in normal fetuses

Prediction of the six landmarks, including the upper/lower lip, middle point of the anterior border of the maxilla/mandible, mentum, and nasion, can directly affect the measurements of facial markers. Consequently, we calculated the mean absolute error between the predicted and manually labeled values. The error values between AI and manual measurements of the six landmarks were 0.20 mm (SD 0.29), 0.15 mm (SD 0.22), 0.20 mm (SD 0.13), 0.20 mm (SD 0.13), 0.17 mm (SD 0.16), and 0.16 mm (SD 0.10), respectively. The collective error of all landmarks was less than 0.2 mm. In addition, the average AI model measurement speed was 0.76 s per image, whereas the speed of manual measurement conducted by a senior sonographer was approximately 2 min per image.

Table 1 compares the measurements of the facial profile markers. In the clinical test set, the AI measurement values for IFA, MNM angle, FMA, FS distance, and PL distance were 82.26° (62.20-102.32), 4.44° (-0.84-9.72), 79.96° (64.94–94.97), 1.41 mm (-1.34-4.22) and 3.17 mm (2.07–4.27), respectively. The manual measurements were 82.28° (61.29-103.28), 4.90° (-0.80-10.60), 80.06° (63.97–96.14), 1.45 mm (-1.69-4.59) and 3.30 mm (2.05–4.53), for the same parameters. The mean measurement deviations were 0.03° (-12.55-12.61), 0.46° (-3.35-4.26), 0.10° (-10.55-10.75), 0.04 mm (-1.86-1.93), 0.12 mm (-0.60-0.85), respectively. Furthermore, the Pearson correlation test demonstrated a strong correlation between the AI and manual measurements for IFA, MNM angle, FMA, FS distance, and PL distance (r = 0.813; r = 0.762; r = 0.767; r = 0.803; r = 0.814, all *P* < 0.001).

**Table 1:** Comparison of the measurements of facial profile markers between the AI and manual measurement

	IFA	MNM angle	FMA	FS distance	PL distance
AI group	82.26° (62.20-102.32)	4.44° (-0.84-9.72)	79.96° (64.94–94.97)	1.41 mm (-1.34-4.22)	3.17 mm (2.07–4.27)
Manual group	82.28° (61.29-103.28)	4.90° (-0.80-10.60)	80.06° (63.97–96.14)	1.45 mm (-1.69-4.59)	3.30 mm (2.05–4.53)
Mean of difference	0.03° (-12.55-12.61)	0.46° (-3.35-4.26)	0.10° (-10.55-10.75)	0.04 mm (-1.86-1.93)	0.12 mm (-0.60-0.85)
r	0.813	0.762	0.767	0.803	0.814
*P*	< 0.001	< 0.001	< 0.001	< 0.001	< 0.001

Data are given as mean (95% CI) or n

*IFA* inferior facial angle, *MNM* maxilla-nasion-mandible, *FMA* facial-maxillary angle, *FS distance* frontal space distance, *PL distance* profile line distance, *AI* artificial intelligence

As illustrated in Table 2, the clinical test set AI and manual measurement group ICCs (95%CI) of IFA, MNM angle, FMA, FS distance, and PL distance were 0.812 (0.780–0.840), 0.760 (0.720–0.795), 0.766 (0.727-0.800), 0.807 (0.775–0.836), and 0.798 (0.764–0.828), respectively. These results indicate strong consistency between AI and manual measurements.

**Table 2:** ICCs of facial profile markers between the AI and manual measurement

	The AI group and manual measurement group	
	ICC	95% CI
IFA	0. 812	0.780–0.840
MNM angle	0. 760	0.720–0.795
FMA	0. 766	0.727-0.800
FS distance	0. 807	0.775-0.836
PL distance	0. 798	0.764–0.828

Data are given as n or 95% CI.

*AI* artificial intelligence, *ICC* intraclass correlation coefficients, *CI* confidence interval, *IFA* inferior facial angle, *MNM* maxilla-nasion-mandible, *FMA* facial-maxillary angle, *FS distance* frontal space distance, *PL distance* profile line distance

**Fig. 4:**
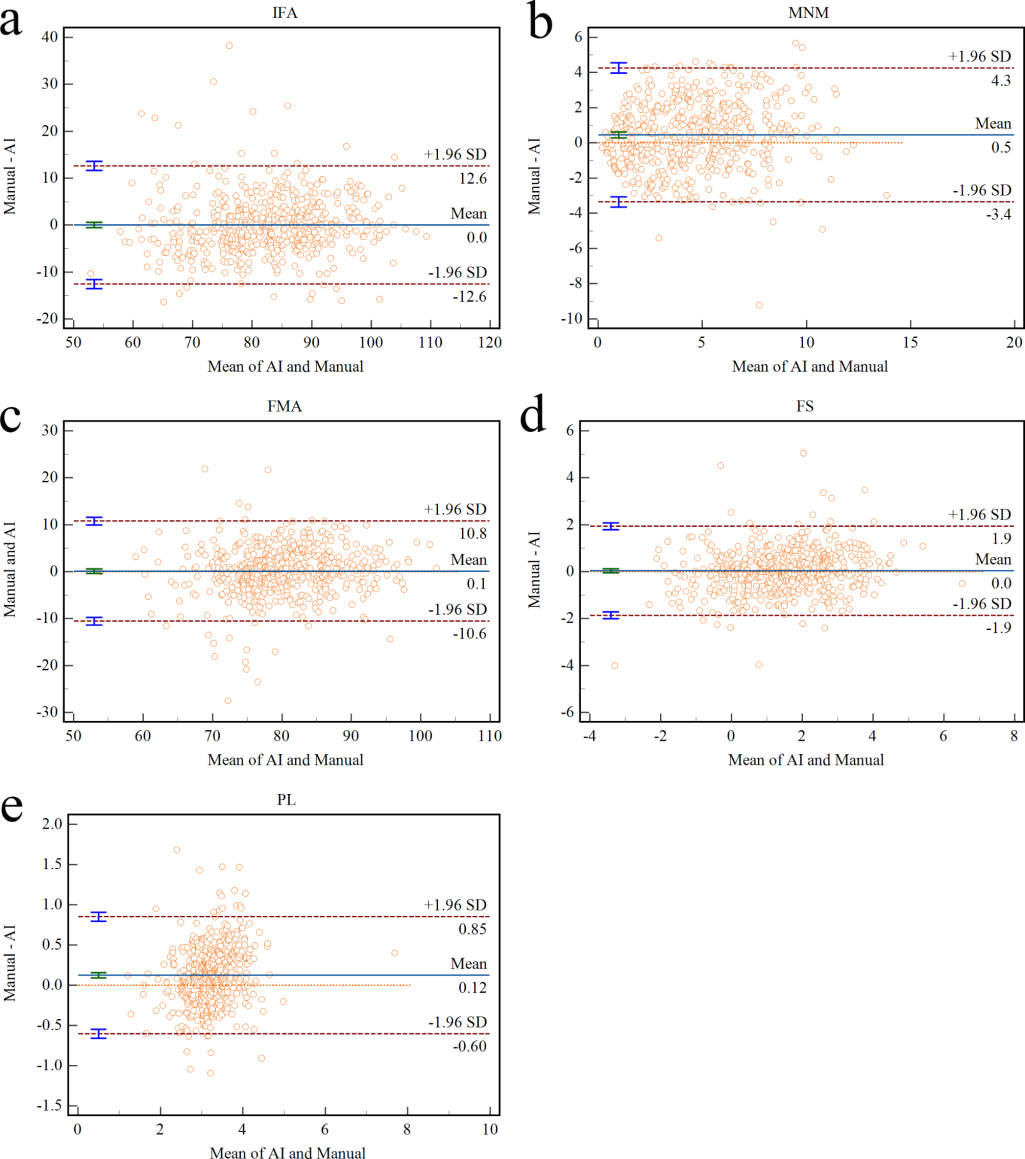
Bland-Altman plots showing the consistency between AI and manual measurements of inferior facial angle (IFA) **(a)**, maxilla-nasion-mandible (MNM) angle **(b)**, facial-maxillary angle (FMA) **(c)**, frontal space (FS) distance **(d)**, and profile line (PL) distance **(e)**. The solid line represents the mean difference between the two measurements, the dotted line represents 95% CI of the difference

**Fig. 5:**
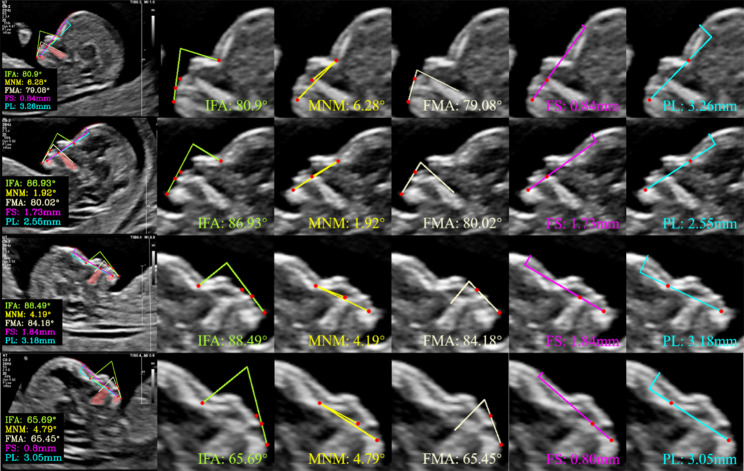
Examples illustrating the measurement results in normal fetal images. The fourth line showed extreme cases of normal fetuses (poor image quality). Each column, from left to right, represents a summary graph of all markers as well as the measurement results of individual markers, including inferior facial angle (IFA), maxilla-nasion-mandible (MNM) angle, facial-maxillary angle (FMA), frontal space (FS) distance, and profile line (PL) distance, respectively

To better visualize the agreement between AI and manual measurements, Bland–Altman diagrams were generated for the five facial markers, as depicted in Fig. 4a-e. Additionally, we selected a subset of the measurement data obtained from normal fetal images, which is presented in Fig. 5. The fourth line in Fig. 5 showed extreme cases of normal fetuses (poor image quality). Each column, from left to right, represents a summary graph of all markers as well as the measurement results of individual markers, including IFA, MNM angle, FMA, FS distance, and PL distance, respectively.

### Performance of AI measurement model for fetal facial markers in detecting fetal abnormalities

**Fig. 6:**
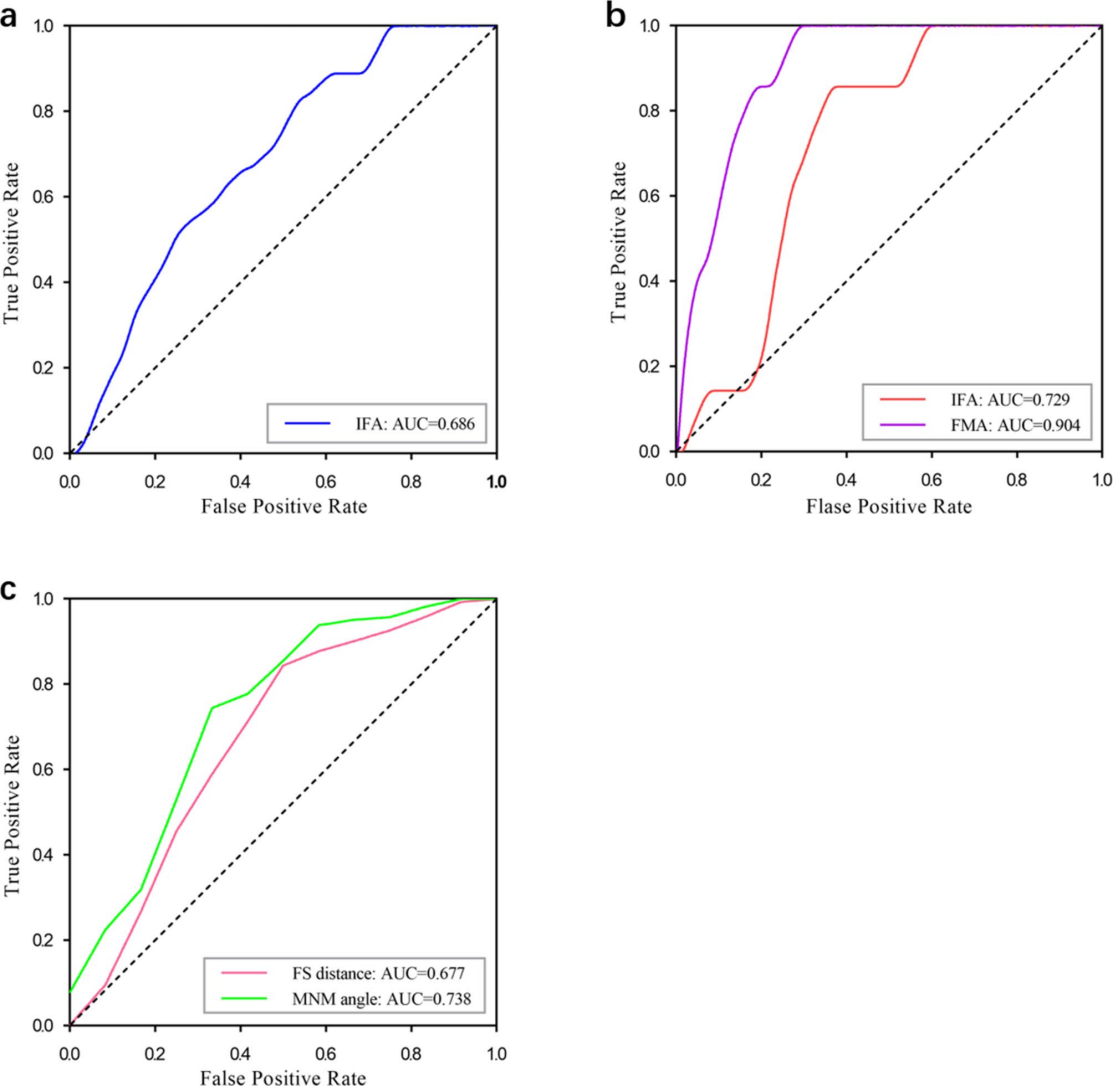
ROC curve of inferior facial angle (IFA) measurements of fetuses with trisomy 21 **(a)**, IFA and facial-maxillary angle (FMA) of fetuses with trisomy 18 **(b)**, maxilla-nasion-mandible (MNM) angle and frontal space (FS) distance of fetuses with cleft lip palate (CLP) **(c)**. Areas under the curves, 95% CI, sensitivity, specificity, and *P* values are given in Table 3

We analyzed the diagnostic value of the AI model in identifying fetal abnormalities by calculating the area under the receiver operating characteristic curve (AUC), presented in Table 3; Fig. 6. Our findings indicated that IFA measurements were clinically significant in identifying trisomy 21 and trisomy 18, with AUCs of 0.686 (95%CI, 0.585–0.788) and 0.729 (95%CI, 0.621–0.837), respectively. FMA measurement exhibited excellent performance in predicting trisomy 18, with an AUC of 0.904 (95%CI, 0.842–0.966). Furthermore, the MNM angle and FS distance demonstrated good predictive values for CLP, with AUCs of 0.738 (95%CI, 0.573–0.902) and 0.677 (95%CI, 0.494–0.859), respectively.

**Table 3:** The diagnostic value of AI model for fetal facial markers in trisomy 21, trisomy 18 and CLP

	Trisomy 21	Trisomy 18		CLP	
	IFA	IFA	FMA	MNM angle	FS distance
AUC	0.686	0.729	0.904	0.738	0.677
95% CI	0.585–0.788	0.621–0.837	0.842–0.966	0.573–0.902	0.494–0.859
Sensitivity	0.833	0.857	1	0.75	0.583
Specificity	0.488	0.666	0.743	0.718	0.813
*P* value	0.006	0.036	0.000	0.004	0.035
Cut-off	83.54	78.56	73.77	6.399	0.05

Data are presented as n or 95% CI.

*IFA* inferior facial angle, *MNM* maxilla-nasion-mandible, *FMA* facial-maxillary angle, *FS distance* frontal space distance, *PL distance* profile line distance, *AUC* area under the receiver operating characteristic curve

However, IFA for CLP, MNM angle for trisomy 21 and trisomy 18, FMA for trisomy 21 and CLP, FS distance for trisomy 21 and trisomy 18, and PL distance for trisomy 21, trisomy 18, and CLP failed to demonstrate any significant predictive value, as evidenced by P-values of 0.421, 0.841, 0.121, 0.66, 0.243, 0.856, 0.571, 0.205, 0.999, and 0.411, respectively.

**Fig. 7:**
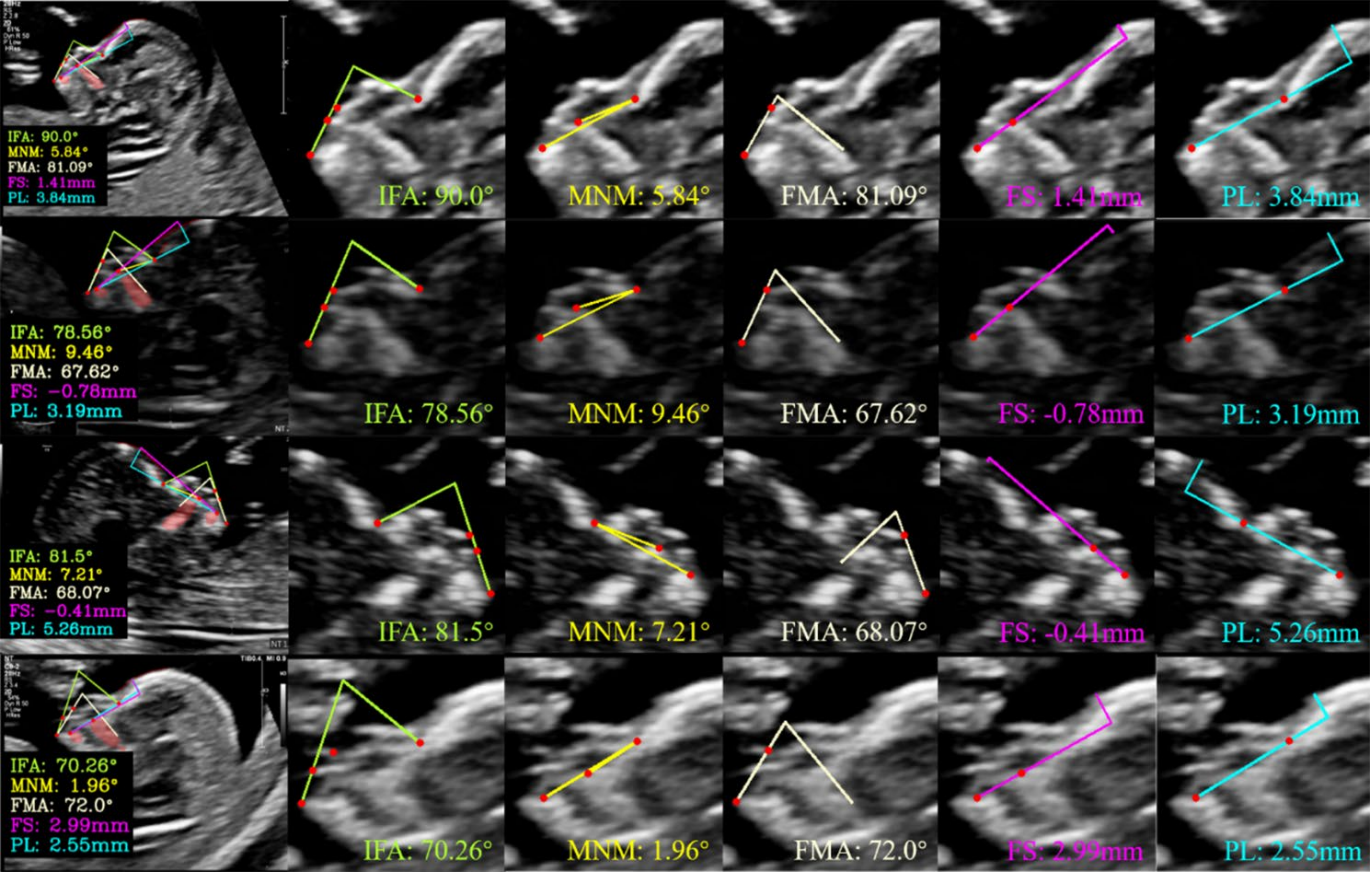
Examples illustrating the measurement results in abnormal fetal images. Each row, from top to bottom, represents the measurement result of AI model for trisomy 21, trisomy 18, CLP, and extreme cases of trisomy 21 (poor image quality) respectively. Each column, from left to right, represents a summary graph of all markers as well as the measurement results of individual markers, including inferior facial angle (IFA), maxilla-nasion-mandible (MNM) angle, facial-maxillary angle (FMA), frontal space (FS) distance, and profile line (PL) distance, respectively

Finally, we present the predictive outcomes of the randomly selected abnormal fetal images in Fig. 7. Each row, from top to bottom, represents the measurement result of AI model for trisomy 21, trisomy 18, CLP, and extreme cases of trisomy 21 (poor image quality) respectively. Each column, from left to right, represents a summary graph of all markers as well as the measurement results of individual markers, including IFA, MNM angle, FMA, FS distance, and PL distance, respectively.

## Discussion

### Principal findings

In this study, we have developed an AI model that utilizes convolutional neural networks to extract multiscale semantic feature sets from fetal faces. The model intelligently measures facial profile markers through the precise segmentation of crucial structures and accurate positioning of landmarks.

To validate the AI model, we conducted a large-scale clinical validation study where facial profile markers were randomly measured in 500 normal fetuses. The ICCs and Bland–Altman analysis indicated good consistency between AI and manual fetal facial profile marker measurements (IFA, MNM angle, FMA, FS distance, and PL distance) during the first trimester, with all ICC values greater than 0.75. Moreover, the Pearson correlation test revealed a statistically significant correlation between the AI and manual measurements (r > 0.75, all *P* < 0.001).

Finally, we investigated the diagnostic value of the AI model for detecting facial abnormalities, and found that the fetal facial markers measured by AI model could effectively detect trisomy 21, trisomy 18, and CLP. In detail, we observed that IFA was accurate in identifying trisomy 21 and trisomy 18, with AUCs of 0.686 and 0.729, respectively. FMA achieved excellent performance in predicting trisomy 18, with an AUC of 0.904. The MNM angle and FS distance exhibited good predictive values for CLP, with AUCs of 0.738 and 0.677, respectively. However, the PL distance remained insignificant in predicting trisomy 21, trisomy 18, and CLP.

### Results in the context of what is known

At present, studies on these facial markers mainly focus on the second and third trimester, we previously demonstrated the feasibility of measuring these markers during the first trimester, with excellent intra- and inter-operator consistency [18]. We further concluded that these markers have certain diagnostic value for fetal abnormalities [24]: IFA had a certain value in the diagnosis for trisomy 21 and trisomy 18; FMA had the excellent accuracy in detecting trisomy 18; MNM angle and FS distance were reliable indicators for screening CLP, which was consistent with this study.

However, manual measurement is laborious and complex, and identifying a technique that can precisely and swiftly measure fetal facial markers is of significant clinical value. In this study, the average inference speed achieved by our model was 0.76 s per image, significantly outperforming manual measurements, which required approximately 2 min per image [24]. Our AI-based approach for measuring facial markers can accelerate the measurement process by a factor of approximately 120. These findings suggest that the AI model for measuring facial markers can facilitate manual measurements, improve work efficiency of sonographers and accelerate early clinical evaluation of fetal prognosis.

### Clinical implications

Ultrasonographic assessment of the fetal face is important in prenatal diagnosis of fetal abnormalities. Fetal facial abnormalities may occur in isolation or may serve as an indication of underlying genetic syndromes. Multisystem syndromes are closely associated with adverse fetal outcomes [25]. Therefore, the diagnosis of facial abnormalities during the first trimester is important to facilitate timely clinical assessment of fetal prognosis. This helps women to make informed decisions regarding their pregnancy, minimizing the waste of social resources.

In the era of non-invasive prenatal testing (NIPT), cell-free fetal DNA (cffDNA) significantly improved the performance of trisomy 21, with a detection rate of > 99% and a false positive rate (FPR) < 0.1% [26]. However, cffDNA was of high cost and it could not be affordable for all pregnant women. Further cost-saving approaches should be explored.

Over the past decade, automatic measurement methods [27] using AI have been implemented to mitigate intra- and inter-operator variation and enhance the precision of ultrasound diagnosis. In this study, AI was successfully applied to FTS to construct a convenient facial markers measurement model, which can effectively predict trisomy 21, trisomy 18, and CLP fetuses, reduce the workload of sonographers and promote the establishment of intelligent medical system.

### Research Implications

This study suggests that utilizing AI model for measuring facial markers to screen for birth defects is a convenient and effective approach. Further verification of our findings, particularly concerning the diagnostic value of IFA in identifying trisomy 21 and FS distance in identifying CLP, requires additional abnormal data. Therefore, a multi-center collaboration with other hospitals could be conducted. Additionally, accurate localization of critical landmarks, including the upper/lower lip, middle point of the anterior border of the maxilla/mandible, mentum, and nasion, is crucial for measuring the mentioned facial markers. Hence, the development of more precise algorithms for locating these landmarks is necessary.

### Strengths and Limitations

The strengths of this study include the large sample of the normal images in comparison with previous study, [18] with a total of 2372 annotated normal fetal images used to develop and validate the AI model. In clinical practice, the traditional manual measurement method requires sonographers to identify facial structures and then measure these markers in the mid-sagittal section, which is arduous, time-consuming, and requires sonographers with exceptional expertise. In contrast, our AI model can measure all facial markers in an average of only 0.76 s and automatically identify trisomy 21, trisomy 18 or CLP. Meanwhile, the AI model improves the interpretability of the entire diagnostic process by automatically locating landmarks and segmenting anatomical structures while ensuring a high degree of consistency with manual measurements.

However, this study has some limitations. First, whilst the majority of mid-sagittal section images could be analyzed effectively, certain unique images were difficult to assess. For example, the tangent at the nasion was a small segmentation area, so measurement was difficult during regional segmentation. Second, the limited number of fetal abnormalities observed may have affected the precision of the model in identifying specific types of abnormalities. Third, this study is mainly related to its retrospective design, prospective studies are required to confirm the accuracy and effectiveness of the AI model in the future.

Finally, in order to verify the effectiveness of different markers in screening abnormalities on a large dataset, our model is trained on high-quality images with quality control. If it is directly applied to clinical scenes, it can lead to false positive and false negative situations due to image quality changes, structural blurriness and other problems, and may be overestimated the consistency of the current AI and manual measurements. However, we believe that in the future, with the input of images, the richness of types, the optimization of the model and the development of prospective studies, our model will show stronger robustness and accuracy.

## Conclusions

In this study, we initially established an AI measurement model for fetal facial profile markers during the first trimester, demonstrating good consistency with manual measurements. This innovative model has significant potential for popularization in FTS. As a convenient and effective tool for early screening for fetal trisomy 21, trisomy 18, and CLP, the tool facilitates early clinical evaluation of fetal prognosis and promotes the cause of reproductive health.

## Data Availability

The datasets and code are not publicly available due to the hospital policy and personal privacy, but are available from the corresponding author on reasonable request.

## Abbreviations

AI: Artificial intelligence
AUC: Area under curve
CLP: Cleft lip and palate
CNNs: Convolutional neural networks
CRL: Crown-rump length
DL: Deep leaning
FMA: Facial maxillary angle
FMF: Fetal Medicine Foundation
FPL: Facial profile line
FS distance: Frontal space distance
FTS: First-trimester scanning
IFA: Inferior facial angle
ICC: Intraclass correlation coefficient
IQR: Interquartile range
ISUOG: International Society of Ultrasound in Obstetrics and Gynecology
LASSO: Least Absolute Shrinkage and Selection Operator
MML: Mandibulo-maxillary line
MNM: Maxilla-nasion-mandible angle
NT: Nuchal translucency
PL distance: Profile line distance
PT: Pre-nasal thickness
ROC: Receiver operating characteristic
SD: Standard deviation
ROI: Region of interest
2D-US: Two-dimensional ultrasound
4D: Four-dimensional
